# Validity and reliability of the EQ-5D-5 L in family caregivers of leukemia patients

**DOI:** 10.1186/s12885-019-5721-2

**Published:** 2019-05-30

**Authors:** Limin Li, Chaojie Liu, Xiuzhi Cai, Hongjuan Yu, Xueyun Zeng, Mingjie Sui, Erwei Zheng, Yang Li, Jiao Xu, Jin Zhou, Weidong Huang

**Affiliations:** 1 First Affiliated Hospital of Harbin Medical University, Harbin, 150001 Heilongjiang China; 2 School of Psychology and Public Health, La Trobe University, Melbourne, 3086 Australia; 3 Harbin center for disease control and prevention, Harbin, 150056 China; 4 School of Health Management, Harbin Medical University, 157 Baojian Road, Nangang District, Harbin, 150086 China

**Keywords:** Leukemia, Family caregiver, EQ-5D-5 L, Validity, Reliability

## Abstract

**Purpose:**

This study aimed to test the validity and reliability of the five-level EuroQol five-dimensional (EQ-5D-5 L) instrument in family caregivers (FCs) of leukemia patients in Heilongjiang of China.

**Methods:**

A cross-sectional survey was conducted on 298 family caregivers (FCs) of leukemia patients from three major cancer centers in the capital city of Heilongjiang province of China. Their dimensional scores of the EQ-5D-5 L were compared with those of the WHOQOL-BREF to test the convergent validity (constructs measuring the same concept) and divergent validity (constructs measuring different concepts) of the EQ-5D-5 L. Repeated surveys were conducted on 271 participants to determine the test–retest reliability of the EQ-5D-5 L.

**Results:**

The four physical dimensions (mobility, self-care, usual activities, and pain/discomfort) of the EQ-5D-5 L had moderate or high correlations with the physical health domain of the WHOQOL-BREF, with a correlation coefficient (r) ranging from 0.459 to 0.559. The anxiety/depression dimension of the EQ-5D-5 L had a high correlation (r = 0.667) with the psychological domain of the WHOQOL-BREF. By contrast, lower but still significant physical-to-psychological correlations were found between the two instruments (r ranging from 0.219 to 0.396). In addition, the EQ-5D-5 L dimensional scores showed no or weak correlations with the environment and social domains of the WHOQOL-BREF (r ranging from 0.016 to 0.207). High test-retest reliability (> 0.7) was evident.

**Conclusion:**

The Chinese version of the EQ-5D-5 L has satisfactory reliability and validity in FCs of leukemia patients. It can be used to elicit utility of health-related quality of life in FCs of leukemia.

## Introduction

Leukemia is one of the common cancers, ranking in top 11 in cancer incidence and top 10 in causes of cancer death [1]. It can happen in adults as well as in children [2]. In 2012, 352,000 patients were diagnosed with leukemia globally, and 265,000 died from leukemia [3]. China reported 75,300 new cases of leukemia and 53,400 deaths of leukemia patients in 2015 alone [4]. The recent medical advancement has improved the prognosis of leukemia significantly. For example, the five-year survival rate of leukemia patients increased from 19.6% in 2003–05 to 25.4% in 2012–15 in China [5], which has resulted in growing prevalence of patients living with leukemia. The Global Burden of Disease 2015 Study (GBD 2015) estimated that the world now has about 2.3 million people living with leukemia [1, 6, 7].

Leukemia is a devastating event not only to the patients but also to their family. Behind each statistic of a new leukemia case is an individual face, accompanied by the faces of family caregivers (FCs) drawn into this singular event. Caring for a patient with leukemia can impose tremendous toll on the physical and emotional health of the family caregivers (FCs) [8, 9]. Previous studies have applied generic instruments such as the 36-Item Short Form Health Survey (SF-36, [10]) and the World Health Organisation Quality of Life-BREF (WHOQOL-BREF) for assessing health-related quality of life (HRQoL) of the FCs. Although these instruments can offer detailed descriptions about HRQoL across a range of domains [11, 12], they are not able to be converted into a single utility index. A single utility index is needed to reflect the overall preference of the public. This is particularly important when one is rated higher in some domains of HRQoL but lower in other domains than other people. Cost-utility analysis has been widely accepted as a useful tool for resource allocation [13, 14]. In recent years, the EQ-5D developed by the EuroQol group has attracted increasing attention for its simplicity and availability of a utility calculation algorithm based on the preference of general public. More than 140 language versions of EQ-5D have been developed [15].

There are two versions of EQ-5D: one asking respondents to rate their experience across three levels (EQ-5D-3 L) and another one across five levels (EQ-5D-5 L). We attempted to use the EQ-5D-5 L in HRQoL assessment on FCs of leukemia patients because it is less likely than the EQ-5D-3 L to show ceiling effect (reaching the maximum possible score) and may be more responsive to monitor small changes, especially in mild conditions [16–18]. However, there is a lack of psychometric evidence to support the validity and reliability of the EQ-5D-5 L in Chinese populations. This study aimed to test the validity and reliability of the EQ-5D-5 L in FCs of leukemia patients. Specifically, we tested the convergent validity, divergent validity, known- groups validity, and test–retest reliability of the EQ-5D-5 L instrument.

## Methods

A cross-sectional questionnaire survey was conducted on FCs of leukemia patients. We selected leukemia for this study for several reasons: (1) Leukemia is one of the top 10 causes of cancer death; (2) Leukemia can happen in adults as well as in children, which presents particular challenges to FCs of patients; (3) High quality of care became critical for improving HRQoL when the prognosis of leukemia is improved significantly.

The patients were recruited from three tertiary hospitals located in the capital city (Harbin) of Heilongjiang province in China. The survey was undertaken between July 2015 and February 2016. The hospitals provided a list of leukemia patients admitted over the period of the survey. Trained interviewers approached the FCs of the patients, seeking informed consent from the FCs for participating in the study. The participating FCs had to be a primary caregiver without receiving any payment, be 18 years or older, and be able to communicate with the interviewers.

**Fig. 1:**
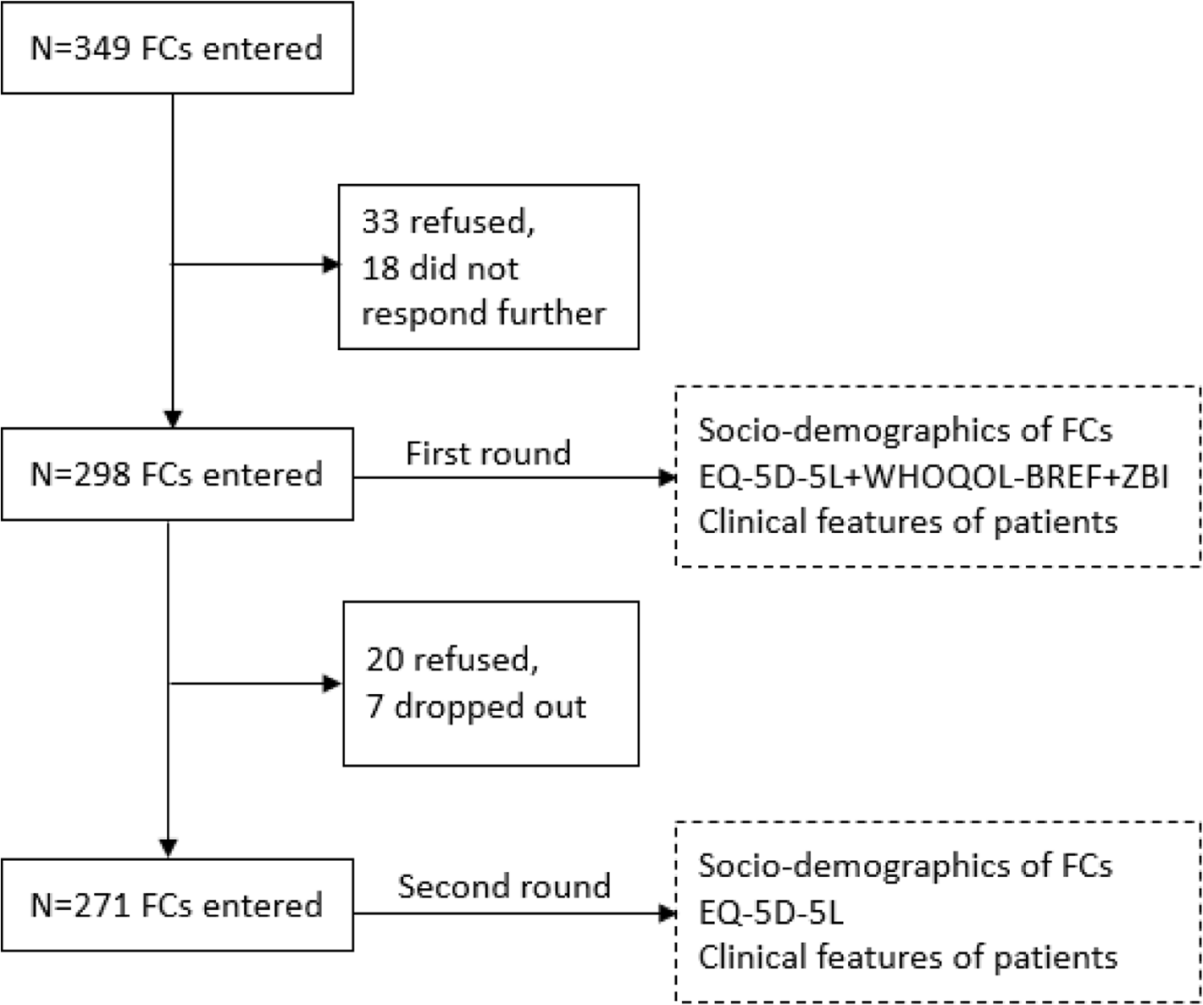
Flow chart of survey

The questionnaire was administered through face to face interviews in a private room in the hospitals where the patients were treated. The interviewers were recruited from postgraduate research students in Harbin Medical University. Training was provided to the interviewers about how to approach potential participants, how to explain the purpose and procedure of this study, how to obtain informed consent, how to assess the eligibility of participants, and how to fill the questionnaire. A total of 349 primary FCs of leukemia patients were approached and 298 (85%) returned a questionnaire that was valid for data analyses (Fig. 1).

### Survey instrument

The questionnaire contained a Chinese version of EQ-5D- 5 L and WHOQOL-BREF measuring HRQoL.

#### EQ-5D-5 l

Participants were asked to rate the problems they experienced on a five-level scale (no problem, mild problems, moderate problems, severe problems, and extreme problems) in relation to five dimensions: mobility, self-care, usual activities, pain/discomfort, and anxiety/depression. The combination of reported problems for each participant was converted into an index score according to the public preference [19]. In addition, the participants were asked to rate their overall health on a visual analogue scale (VAS) ranging from 0 (worst health) to 100 (best health). Each participant was asked to complete the EQ-5D-5 L twice by the same interviewer, 24 h apart. But participants could choose to complete only one or both (91%). This resulted in a final sample size of 271 for the repeated survey.

#### WHOQOL-BREF

This is a well-established generic instrument measuring HRQoL, which has been validated in China [9, 20]. It contains 26 items, measuring physical health (7 items), psychologic health (6 items), social relationships (3 items) and environment (8 items), as well as perceived overall quality of life and general health (2 items) [21]. Each domain has a score ranging from 0 to 20, with a higher score denoting higher HRQoL.

The questionnaire also collected data in relation to the clinical feature of the leukemia patients [9, 22, 23] (gender, age, types of leukemia, and performance status) and the socio-demographic characteristics of the FCs (gender, age, ethnicity, relationship to patient, educational attainment, marital status, employment, religious belief, and annual household income) and burden of caring for patients. Patient characteristics have significant implications on the burden of care of FCs.

#### Type of leukemia

Four types of leukemia were included in this study: acute myelogenous leukemia (AML), acute lymphoblastic leukemia (ALL), chronic myelogenous leukemia (CML), and chronic lymphoblastic leukemia (CLL).

#### Performance of patients

The performance status of the patients was measured by the Eastern Cooperative Oncology Group (ECOG) scale [24]. Clinicians (doctors or nurses) recorded a grade for each patient on the day of the initial survey along a six-point spectrum, with one end (0) indicating fully functional and the other end (4) indicating completely disabled and confined to bed/chair.

#### Burden of care

The burden of caring for leukemia patients was measured by the Zarit Burden Inventory (ZBI). The ZBI has been validated in previous studies in China [25]. It contains 22 items [26]. Respondents rated on a 5-point Likert scale (ranging from 0 being “rarely” to 4 being “always”) for each item. The scores were summed up (ranging from 0 to 88), with a higher score indicating a higher burden of care. The burden of caring for leukemia patients was categorized into four groups: little or no burden (0–20), mild to moderate burden (21–40), moderate to severe burden (41–60), and severe burden (61–88).

### Data analysis

We described the characteristics of FCs, including the characteristics of patients they cared for. The proportion of FCs reporting any problems in each EQ-5D-5 L dimension and EQ-5D-5 L index were presented. The EQ-5D-5 L utility values used in the study were derived from the recently developed Chinese EQ-5D-5 L value set [19], which indicates the preference of the general Chinese population on various health states. The EQ-5D-5 L utility index ranged from − 0.391 to 1, and higher values indicated better health status.

The reliability of the EQ-5D-5 L was determined by the repeated tests. We calculated Cohen’s Kappa coefficient for each dimension of the EQ-5D-5 L and intraclass correlation coefficient (ICC) for the index score and the VAS score. A coefficient value of over 0.60 indicates substantial agreement, while over 0.80 indicates almost perfect agreement [27, 28].

We examined the convergent validity and divergent validity of the EQ-5D-5 L using Spearman’s rank correlation analyses. A correlation coefficient between 0.1 and 0.29 was considered as weak, 0.30–0.49 as moderate, and above 0.5 as strong associations [29]. Basing on the existing literature [30], we tested the following hypotheses:

1. EQ-5D-5 L scores have moderate to strong correlations with WHOQOL-BREF scores measuring similar concepts: for example between the physical health domain of the WHOQOL-BREF and the mobility, self-care, usual activity, and pain/discomfort dimensions in the EQ-5D-5 L; and between the psychological health domain of the WHOQOL-BREF and the anxiety/depression dimension in the EQ-5D-5 L *(Convergent validity).*
2. Reported health problems increase with ZBI scores; whereas EQ-5D-5 L index and VAS scores decrease with ZBI scores *(Convergent validity).*
3. The domains measuring physical health have weak correlations with those measuring psychological/mental health and vice versa compared with those measuring similar concepts between the EQ-5D-5 L and the WHOQOL-BREF *(Divergent validity).*
4. EQ-5D-5 L dimensional scores have weak or no correlations with the environment and social domains of the WHOQOL-BREF *(Divergent validity).*
5. The environment and social domains of the WHOQOL-BREF have a stronger correlation with the VAS score of the EQ-5D-5 L than with the index score of the EQ-5D-5 L, because VAS ratings are likely to include a consideration of environment and social factors which is absent from the index score calculation algorithm *(Divergent validity).*

There exist health related gradients in the EQ-5D-5 L index and VAS scores of the FCs: the EQ-5D-5 L index and VAS scores are associated (Kruskal–Wallis tests) with the health of both patients (measured by ECOG) and FCs (perceived overall health) (*Known-group validity*).

All data analyses were carried out using the Statistical Package for Social Sciences (SPSS) version 20.0. A *p* value less than 0.05 was considered statistical significant.

## Results

### Characteristics of study participants

The participating FCs had a mean age of 40 years. About 55% were female. The majority were either spouse or parent of the patients (80%), well educated (87% completed middle schools), and employed (77%). The patients they cared for were predominantly AML (53%) and ALL (31%) patients and had a mean age of 34 years. On average, the participating FCs experienced moderate burden of care, with a mean ZBI score of 40.60 (Table 1). A high percentage of the FCs reported problems (all levels inclusive): 26% in mobility, 26% in self-care, 30% in usual activities, 54% in pain/discomfort, and 61% in anxiety/depression. The mean score of EQ-5D-5 L index and EQ-VAS were 0.813 (±0.221) and 73.56 (±16.13), respectively. The FCs of leukemia patients had an average score in the four domains of WHQQOL-BREF: 12.72 ± 2.70 for physical, 12.23 ± 3.38 for psychological, 13.11 ± 3.69 for social and 11.34 ± 3.04 for environment (Table 1).

**Table 1:** Characteristics of family caregivers and their care recipients

Characteristics of family caregivers	Initial survey (*n* = 298)	Repeated survey (*n* = 271)
Gender (% of female)	163 (54.7%)	150 (55.4%)
Age (years, mean ± SD)	41.08 (±10.80)	40.90 (±10.79)
Ethnicity (% of Han)	288 (96.6%)	268 (97.0%)
Relationship to patient (n, %)		
Spouse	107 (35.9%)	94 (34.7%)
Parent	132 (44.3%)	123 (45.4%)
Child	43 (14.4%)	40 (14.8%)
Other	16 (5.4%)	14 (5.2%)
Level of education (n, %)		
No more than primary school	37 (12.4%)	35 (12.9%)
Middle or high school	196 (65.8%)	176 (64.9%)
University	65 (21.8%)	60 (22.1%)
Marital status (n, %)		
Married	280 (94.0%)	255 (94.1%)
Other	18 (6.0%)	16 (5.9%)
Employment (n, %)		
Employed	229 (76.8%)	208 (76.8%)
Retired	21 (7.1%)	18 (6.6%)
Unemployed	48 (16.1%)	45 (16.6%)
Religious belief (n, %)		
No	254 (85.2%)	230 (84.9%)
Yes	44 (14.8%)	41 (15.1%)
Annual household income (Yuan)		
≤ 40,000	161 (54.0%)	152 (56.1%)
40,001-79,999	128 (43.0%)	111 (41.0%)
≥ 80,000	9 (3.0%)	8 (3.0%)
ZBI (mean ± SD)	40.60 (±16.29)	–
Little or no	13.90 (±3.35)	–
Mild to moderate	30.74 (±6.16)	–
Moderate to severe	47.88 (±5.14)	–
severe	66.91 (±6.43)	–
WHOQOL-BREF (mean ± SD)		
Physical score	12.72 (±2.70)	–
Psychological score	12.23 (±3.38)	–
Social score	13.11 (±3.69)	–
Environment score	11.34 (±3.04)	–
EQ-5D-5 L index (mean ± SD)	0.813 (±0.221)	–
EQ-VAS (mean ± SD)	73.56 (±16.13)	–
Characteristics of care recipients		
Gender(% of female)	157 (52.7%)	142 (52.4%)
Age (years, mean ± SD)	35.34 (±20.78)	34.88 (±21.10)
Types of leukemia (n, %)		
ALL	91 (30.5%)	86 (31.7%)
AML	158 (53.0%)	140 (51.7%)
CLL	8 (2.7%)	8 (3.0%)
CML	41 (13.8%)	37 (13.7%)

### Test-retest reliability

**Fig. 2:**
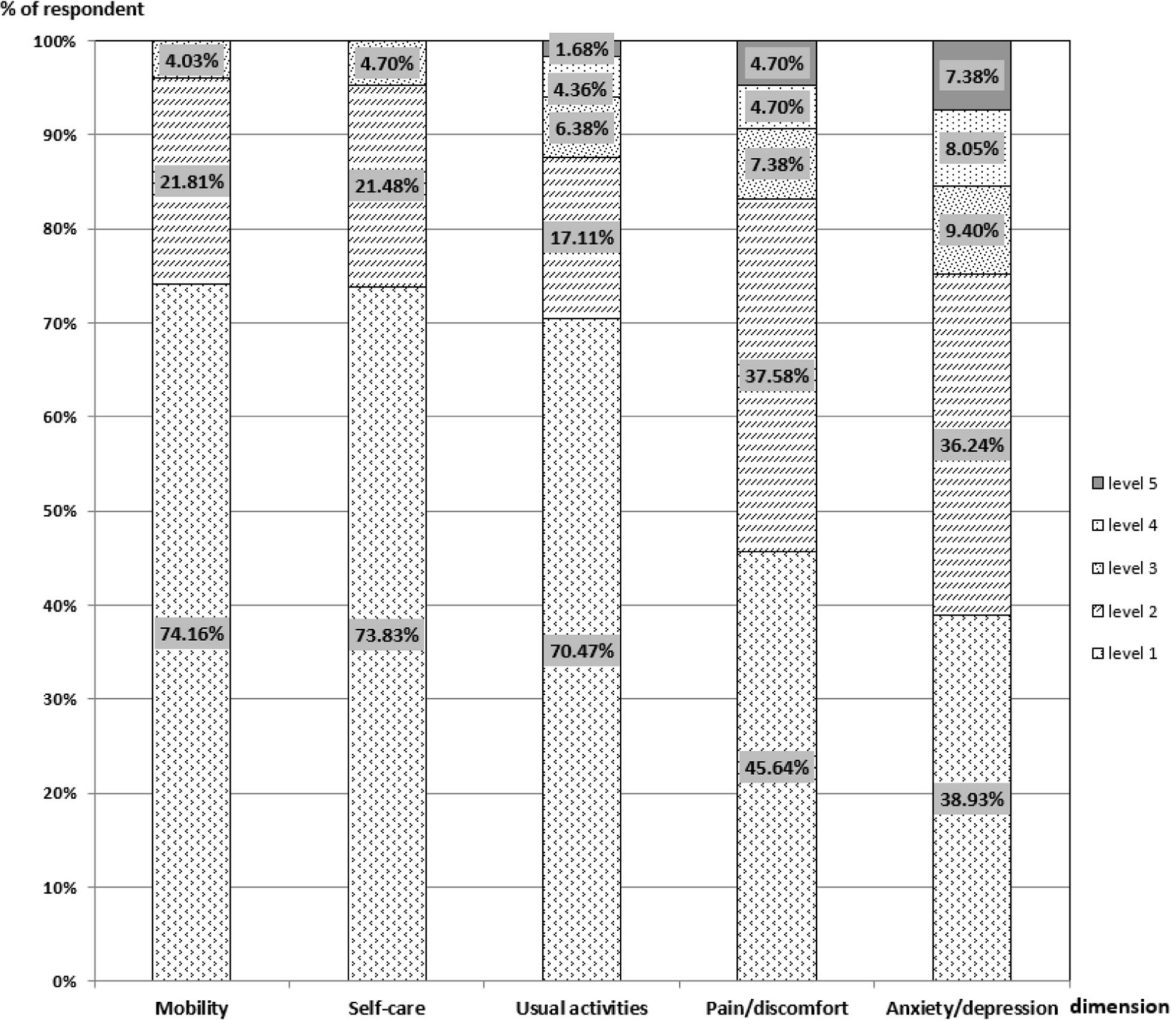
Distribution of health problems across the five dimensions of the EQ-5D-5 L. Note: level 1 *no problems*, level 2 *mild problems*, level 3 *moderate problems*, level 4 *severe problems*, level 5 *extreme problems*

High test-retest reliability was found. The agreements in reported problems across the five dimensions of the EQ-5D-5 L ranged from 86.35% (Anxiety/depression) to 94.10% (mobility) (Fig. 2), with a Cohen’s kappa coefficient exceeding 0.80. The ICC reached 0.987 and 0.865 for the utility index and VAS scores, respectively (Table 2).

**Table 2:** Test-retest reliability of the EQ-5D-5 L (n = 271)

Dimension	Kappa statistics (95% CI)	Agreement rate (%)
Mobility	0.852 (0.777–0.914)	94.10
Self-care	0.794 (0.712–0.876)	91.88
Usual activities	0.833 (0.766–0.896)	91.88
Pain/discomfort	0.846 (0.791–0.897)	90.04
Anxiety/depression	0.804 (0.746–0.865)	86.35
Intraclass correlation coefficient (95% CI)		
EQ-5D-5 L index score	0.987 (0.983–0.991)	
VAS score	0.865 (0.826–0.895)	

### Convergent validity

The physical health domain of the WHOQOL-BREF had high correlations with the mobility (r = − 0.559) and self-care (r = − 0.528) dimensions of the EQ-5D-5 L, and moderate correlations with the usual activities (r = − 0.485) and pain/discomfort (r = − 0.459) dimensions of the EQ-5D-5 L. Similarly, high correlation (r = − 0.667) was found between the psychological domain of the WHOQOL-BREF and the anxiety/depression dimension of the EQ-5D-5 L (Table 3). With the increase in burden of care, reported problems in the five dimensions of the EQ-5D-5 L increased, and the utility index and VAS scores decreased (Table 3). Therefore, hypotheses (1, 2) were supported.

**Table 3:** Correlations of EQ-5D-5 L dimensional, index, and VAS scores with WHOQOL-BREF domain scores and ZBI

	Mobility	Self-care	Usual activities	Pain/discomfort	Anxiety/depression	EQ-5D-5 L VAS	EQ-5D-5 L Index
WHOQOL-BREF							
Physical	−0.559^**^	−0.528^**^	−0.485^**^	−0.459^**^	−0.396^**^	0.529^**^	0.614^**^
Psychological	−0.302^**^	−0.283^**^	−0.219^**^	− 0.336^**^	− 0.667^**^	0.423^**^	0.532^**^
Social	− 0.182^**^	− 0.161^**^	− 0.207^**^	− 0.191^**^	− 0.167^**^	0.331^**^	0.249^**^
Environment	− 0.016	− 0.094	− 0.107	− 0.129^*^	− 0.195^**^	0.300^**^	0.186^**^
ZBI	0.304^**^	0.232^**^	0.184^**^	0.238^**^	0.318^**^	−0.393^**^	−0.394^**^

** *P* <  0.01, * *p* <  0.05

### Divergent validity

The physical-to-mental or psychological-to-physical dimensional correlations (0.219–0.396) were weaker, but still significant, compared with the physical-to-physical and psychological-to-mental dimensional correlations (0.459–0.667) between the EQ-5D-5 L and the WHOQOL-BREF (Table 3). The environmental domain of the WHOQOL- BREF had no significant correlations with the mobility, self-care and usual activities dimensions and weak correlations (0.129–0.195) with the pain/discomfort and anxiety/depression dimensions of the EQ-5D-5 L. Weak correlations (0.161–0.207) were found between the social domain of the WHOQOL-BREF and the five domains of the EQ-5D-5 L (Table 3). The environment (r = 0.300) and social (r = 0.331) domains of the WHOQOL-BREF had moderate correlations with the VAS score of the EQ-5D-5 L, stronger than with the utility index score of the EQ-5D-5 L (0.186 for environment and 0.249 for social domains, respectively) (Table 3). Therefore, hypotheses (3, 4, 5) were supported.

### Known group validity

As expected, the FCs who perceived poorer overall health showed significantly lower utility index and VAS scores in the EQ-5D-5 L. Similarly, the FCs who cared for patients with a higher ECOG performance score had lower utility index and VAS scores in the EQ-5D-5 L (Table 4).

**Table 4:** Variations in EQ-5D-5 L index and VAS scores of family caregivers along with self-perceived health of caregivers and performance of care recipients

Health condition	N	EQ-5D index		EQ-VAS	
Self reported overall health					
Very good	37	0.999	0.007	89.57	7.25
Good	33	0.973	0.039	84.58	9.13
Fair	89	0.871	0.161	76.58	13.96
Poor	85	0.787	0.163	69.32	12.44
Very poor	54	0.536	0.252	57.57	15.62
P*		< 0.001		< 0.001	
Patient performance (ECOG)					
0	64	0.962	0.102	84.52	9.80
1	153	0.831	0.186	74.15	15.27
2	55	0.683	0.239	66.78	15.49
3+	26	0.619	0.294	57.50	15.29
P*		< 0.001		< 0.001	

* p values with Kuskal Wallis Tests

## Discussion

This study has provided empirical evidence to substantiate the reliability and validity of the EQ-5D-5 L. Such a study is important simply because the EQ-5D-5 L has been widely used in over 100 countries across a range of disease conditions and general populations including in China [31]. Although its psychometric properties have only been reported in a few populations [32–34], to the best of our knowledge, the current study was the first to evaluate formally validity and reliability of EQ-5D-5 L among FCs of leukemia patients.

The study showed that the EQ-5D-5 L is highly reliable in the FCs of leukemia patients. All of the five dimensions as well as the utility index and VAS scores exhibited very good test-retest reliability, with Kappa and ICC values equaling or exceeding 0.8. A single study on psychometric properties of the EQ-5D-3 L in caregivers of autistic children did not test test–retest reliability of the EQ-5D-3 L instrument because of its design deficiencies [23]. Such a degree of agreements in the current study is higher compared with the findings of studies conducted elsewhere in various patients [32, 35]. This may be a result of several reasons: first, the repeated test was conducted 24 h after the initial survey and the interval length is shorter compared to the other studies; second, FCs are less likely to experience changes in HRQoL than patients who are undergoing treatments; third, we maintained the same interviewers for repeated tests and avoided inter-interviewer variations. Previous studies (test by interviewers and retest by telephone or mail) demonstrated that the presence of interviewers and different administration methods can exert some influence on the results of a questionnaire survey [32, 36].

The study confirmed the convergent validity and the divergent validity of the EQ-5D-5 L through a comparison with the WHOQOL-BREF. Both the WHOQOL-BREF and EQ-5D questionnaires are commonly used HRQoL tools for the FCs of patients. One of the advantages of the EQ-5D-5 L over the WHOQOL-BREF is that it can be used to measure utility values which make cost-utility analysis and economic evaluations of healthcare interventions possible. Two of domains (physical and psychological) of the WHOQOL-BREF can find an equivalent match in the EQ-5D-5 L. Although respondents may include a consideration of the social and environment factors in their VAS ratings, no equivalent constructs can be found in the EQ-5D-5 L for the social and environment domains of the WHOQOL-BREF. These similarities and differences enabled us to test the convergent validity and divergent validity of the EQ-5D-5 L. In addition, one of the strengths of this study rests on the characteristics of the study participants. For FCs of leukemia patients, we were able to directly estimate their burden of care and test its association with HRQoL. As expected, we revealed some moderate correlations between the EQ-5D-5 L scores and the ZBI scores. These findings are consistent with a previous study [23]. All of the evidence points to a perfect fitness with the conceptual hypotheses. However, the correlation between mobility of EQ-5D-5 L and ZBI score was moderate. This finding was not consistent with previous study [23] and our expectation, and should be further studied in the future.

In addition, we were also able to test gradient changes in the EQ-5D-5 L utility index and VAS scores of the FCs along with the performance of the leukemia patients who they cared for and the perceived overall health of themselves: both were confirmed. These findings may indicate that the EQ-5D-5 L has a strong discriminatory power between known groups of FCs. Previous studies have produced different conclusions about the connection between HRQoL of FCs and the functional status of the patients they cared for. A study in the US detected differences of the Quality of Well-Being (QWB) in the FCs caring for children with spina bifida with different levels of functioning [37]. But another study also in the US failed to establish a connection between the Health Utility (HUI-II) of the FCs and the severity of Alzheimer’s disease of their care recipients [38]. The authors argued that the generic instruments may not be sensitive enough to capture the potential differences. We found the EQ-5D-5 L is sensitive in terms of distinguishing preference scores among caregivers by the level of functioning of leukemia patients. This finding is similar to a study on caregiver of autism with EQ-5D-3 L [23]. In addition, this study also found that FCs who were reporting poorer health tend to have lower EQ-5D-5 L and EQ-VAS scores. This also further confirmed the EQ-5D known-groups validity found in previous validation study [30].

There are several limitations in this study that should be acknowledged. This study was not conducted in a representative sample of participants. It is also limited to FCs of leukemia patients. Further studies in a variety of populations are needed in the future. The cross-sectional design prevented us from testing causal relationships. To avoid potential changing conditions, we performed repeated tests in a short period of time. We cannot exclude the possibility of influence of the initial survey on the repeated survey.

## Conclusion

The Chinese version of EQ-5D-5 L has satisfactory reliability and validity in FCs of leukemia patients. Given that there is no FCs-specific instrument measuring health utility, the EQ-5D-5 L presents a psychometrically sound instrument for researchers to undertake health economic studies, such as cost-utility analyses.

## Abbreviations

ALL: Acute lymphocytic leukemia
AML: Acute myelogenous leukemia
CLL: Chronic lymphocytic leukemia
CML: Chronic myelogenous leukemia
ECOG: Eastern Cooperative Oncology Group
EQ-5D: EuroQol five-dimensional
FCs: Family caregivers
HRQoL: Health-related quality of life
ICC: Intraclass correlation coefficient
VAS: Visual analogue scale
WHOQOL-BREF: World Health Organisation Quality of Life-BREF
ZBI: Zarit Burden Inventory
